# Supplementary material for: HIVprotI: an integrated web based platform for prediction and design of HIV proteins inhibitors
Source: J Cheminform. 2018 Mar 9;10:12. doi: 10.1186/s13321-018-0266-y (PMC5845081; doi:10.1186/s13321-018-0266-y)
Supplement: Supplementary file 2 — Additional file 2. Source code of HIVProtI web server. [file 13321_2018_266_MOESM2_ESM.zip › HIVprotI_Source-code/hivprotI/weka-3-6-6/doc/weka/classifiers/functions/SPegasos.html]

SPegasos


---


|  |  |  |
| --- | --- | --- |
| **Overview** \| **Package** \| **Class** \| **Tree** \| **Deprecated** \| **Index** \| **Help** \| **Weka's home** | |  |
| **PREV CLASS**   **NEXT CLASS** | **FRAMES**    **NO FRAMES**     **All Classes** |  |
| SUMMARY: NESTED \| FIELD \| CONSTR \| METHOD | DETAIL: FIELD \| CONSTR \| METHOD |  |


---


## weka.classifiers.functions Class SPegasos

```
java.lang.Object
  weka.classifiers.Classifier
      weka.classifiers.functions.SPegasos
```

**All Implemented Interfaces:**: java.io.Serializable, java.lang.Cloneable, UpdateableClassifier, CapabilitiesHandler, OptionHandler, RevisionHandler, TechnicalInformationHandler

---

``` public class SPegasos extends Classifier implements TechnicalInformationHandler, UpdateableClassifier, OptionHandler ```

Implements the stochastic variant of the Pegasos (Primal Estimated sub-GrAdient SOlver for SVM) method of Shalev-Shwartz et al. (2007). This implementation globally replaces all missing values and transforms nominal attributes into binary ones. It also normalizes all attributes, so the coefficients in the output are based on the normalized data. For more information, see
